# Creating demand for unmet needs: Agile Storytelling

**DOI:** 10.3389/frhs.2024.1376695

**Published:** 2024-10-31

**Authors:** Jade Mehta, Emily Long, Vidhur Bynagari, Fereshtehossadat Shojaei, Fatemehalsadat Shojaei, Andrew R. W. O’Brien, Malaz Boustani

**Affiliations:** ^1^ Center for Health Innovation and Implementation Science, School of Medicine, Indiana University, Indianapolis, IN, United States; ^2^ Department of Informatics, Luddy School of Informatics, Computing, and Engineering, Indiana University, Bloomington, IN, United States; ^3^ Department of Medicine, School of Medicine, Indiana University, Indianapolis, IN, United States; ^4^ Center for Aging Research, Regenstrief Institute, Inc., Indianapolis, IN, United States; ^5^ Sandra Eskenazi Center for Brain Care Innovation, Eskenazi Health, Indianapolis, IN, United States

**Keywords:** Agile science, implementation science, sickle cell disease, demand, storytelling

## Abstract

**Introduction:**

The translational gap from the discovery of evidence-based solutions to their implementation in healthcare delivery organizations derives from an incorrect assumption that the need for change among executive, administrative, or clinical personnel is the same as the demand for change. For sickle cell disease (SCD), implementation of evidence-based guidelines is often delayed or obstructed due to lack of demand. This challenge allows for the persistence of resource limitations and care delivery models that do not meet the community's unique needs. Agile Storytelling is a process built on the scientific foundations of behavioral economics, complexity science, and network science to create local demand for the implementation of evidence-based solutions.

**Methods:**

Agile Storytelling includes a design phase and a testing phase. The design phase converts the evidence-based solution into a minimally viable story of a hero, a villain, struggle, drama, and a resolution. The testing phase evaluates the effectiveness of the story via a series of storytelling sprints in the target local healthcare delivery organization. The efficacy of Agile Storytelling was tested in an iterative *n*-of-1 case study design.

**Results:**

Agile Storytelling was used in a large, urban, healthcare system within the United States to facilitate implementation of national SCD best-practice guidelines. After repeated failures attempting to use national and local data regarding the high societal need to hire a SCD-specific social worker, an Agile change conductor using Agile Storytelling was able to create demand for the new position within a week. This decision has ultimately improved patient outcomes and led to the adoption of a specialized collaborative care team for SCD within the health network.

**Discussion:**

Agile Storytelling can lead to structured, effective, and informed storytelling to create local demand within healthcare delivery organizations.

## 1 Introduction

One of the major barriers to the implementation and diffusion of evidence-based care solutions is the lack of demand for the adoption of such solutions (1–3). Demand for adoption is not the same as the need for adoption amongst health personnel or patients. There is no shortage of need for change within health systems. In America, 72% of the public report being dissatisfied with chronic care, 57% of physicians believe their ability to provide high-quality care has decreased, and 76% of nurses indicate working conditions interfere with their delivery of quality care (4). Reasons for discontent range from flaws in system design, to rapid changes in technology, to worker shortages, to poor accommodation of diverse patient populations (4). Internationally, the World Health Organization reports major health needs, especially in low- and lower-middle income countries where constant under-investment and health workforce shortfalls persist (5–8). As a result, the needs of patients, professionals, and administrators in health systems are not being met (4, 5, 9). And yet, need alone is not enough to motivate individual behavior change or system-wide reform, whereas demand represents the desire or motivation for a specific outcome and is the primary driver behind behavioral change (10–14). Demand is a personal investment of time, social, or financial capital in adopting certain solutions (1–3, 15). Building sufficient demand for the adoption of evidence-based care solutions within a complex adaptive healthcare delivery network requires executive, administrative, and clinician investment of their own time, social, or financial resources (1–3, 15). Despite the large implementation gap, many in the scientific community assume that the presence of high societal need and data alone can create demand (16–25). Ironically, empirical data does not support such a belief (22–25).

Sickle cell disease (SCD) is a chronic genetic condition affecting over 100,000 people in the United States, mainly African American and Hispanic populations (26). Outside of the United States, the disability burden of SCD is highest in sub-Saharan Africa and India, with sub-Saharan Africa seeing the highest increase in SCD incidence and prevalence over the past 20 years while rates have been stable or declined in most other regions (27). The acute and chronic complications of SCD can disproportionately overwhelm health systems in lower- and middle-income countries, where limited high quality and long-term access to care are associated with higher mortality rates (27, 28). Within the United States, the quality of healthcare services for people with SCD is suboptimal, with an uneven distribution of high-quality care across geographically diverse institutions (26, 29–34). For example, from 2010 to 2015, there were over 1.4 million emergency department visits from SCD patients due to pain crises (35). However, only 46% of these vulnerable patients received timely pain interventions and only 54% believed that clinicians cared about them, exacerbating minority distrust of medical institutions (26, 35–37). The National Heart Lung and Blood Institute (NHLBI) has created guidelines on providing the most evidence-based care for individuals with SCD. Nevertheless, widespread adoption of these guidelines has been minimal (26, 38–41). The World Health Organization SICKLE package provides international guidance on SCD management, but compliance with guidelines and standard-care practices continue to fall short (42–44).

The practice of storytelling has also long been recognized as a valuable tool for increasing the adoption and diffusion of health-promoting behaviors and humanistic practices (45, 46). Systematic evidence reviews of storytelling usage in healthcare show positive implementation benefits with better patient satisfaction and care (47, 48). By definition, storytelling is a narrative that uses plot and characters to create new insights for the teller and listener of the story (49, 50). Storytelling is a low-cost intervention for disseminating information and behavioral change that is universally embedded in cultures and scholarship (45, 51, 52). For example, a study from the Center for American Indian Research and Education illustrates how the historical significance of stories makes storytelling adapted to American Indian traditions a promising intervention for increasing health education and positive lifestyle alterations (53). Other studies emphasize the importance of storytelling in non-Western societies as a primary way to change health behaviors, knowledge, and perception by grounding them in their local setting (54). Finally, digital storytelling interventions have been successful in reaching a range of audiences from individuals in rural South Africa to low-income youth in South Wales to Latino adults in the United States, addressing health promoting behaviors for HIV, Type 2 Diabetes, HPV, cancer, and food insecurity (55). At the individual level, storytelling can reduce resistance to change, resulting in population shifts in public knowledge, attitude, and behavior (45, 56). Because large numbers tend to be perceived as cardinal values while small numbers are tracked as objects through time and space, humans empathize more with a small number of detailed cases than with large statistics (57, 58). Storytelling also increases comprehension and recall compared to facts (59, 60). While data is crucial in confirming the need for evidence-based practices, storytelling drives the demand for system-wide change.

**Figure 1:**
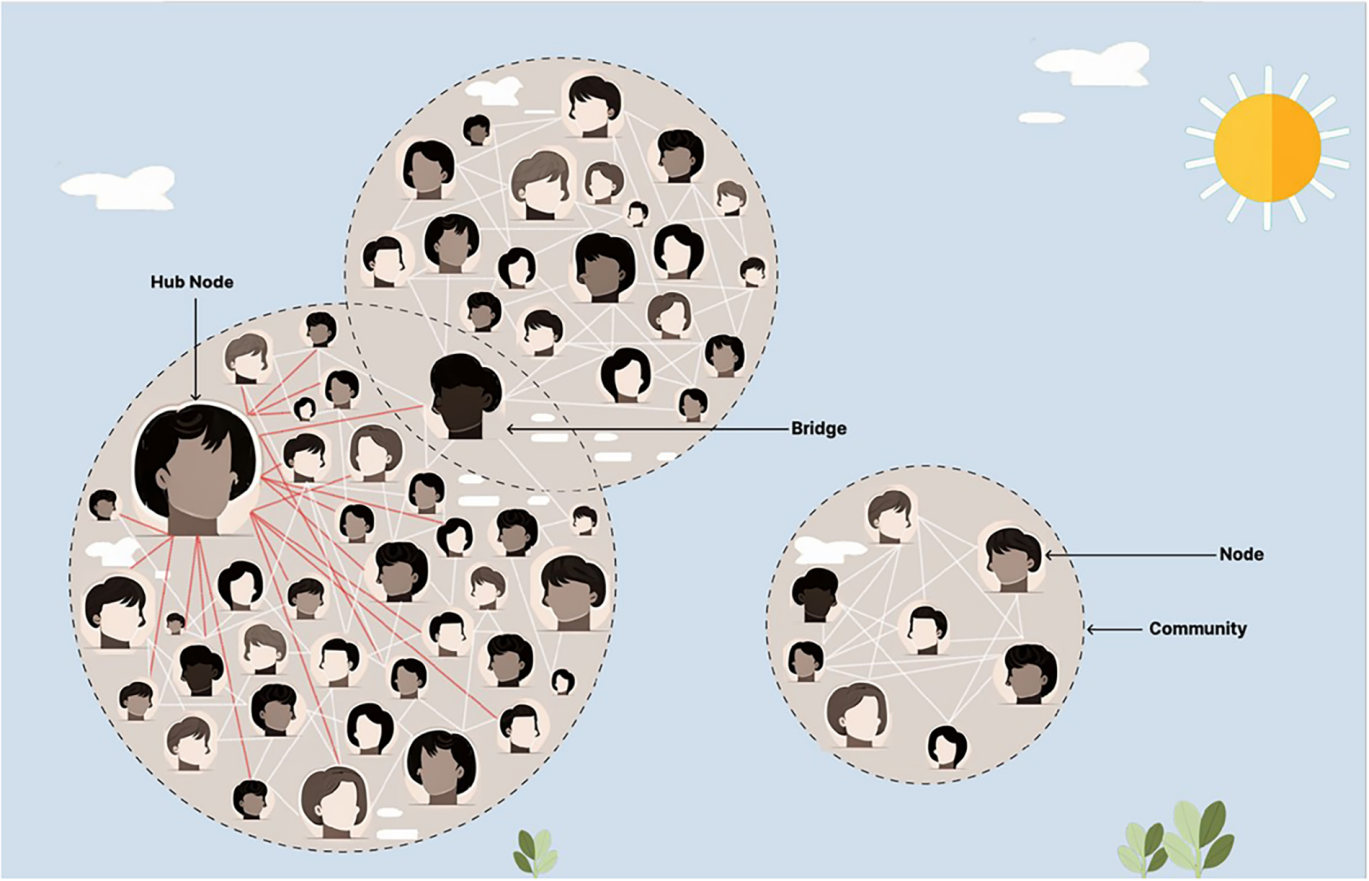
Mapping a complex adaptive human network.

Agile Science is a field within the translational sciences that has been shown to increase the innovation, implementation, and diffusion of evidence-based practices, like that of storytelling (1–3, 61, 62). Agile Science reliably aids in the creation of behavior-change interventions, optimizes project management processes, helps repurpose existing to solutions to have a broader impact, and has been shown to shift scientific norms across a diverse number of disciplines (63–82). Feasibility testing methods for Agile Science often use iterative *n-*of-1 clinical trials to promote individualized and adaptive medicine (66, 83–88). Agile Science uses insights from behavioral economics, complexity science, and network science to understand, predict, and steer a complex adaptive human network (1–3). Behavioral economics investigates the psychological, cognitive, emotional, cultural, and social factors of decision-making, particularly in situations with high uncertainty, variability, and dynamic interdependence (89–93). In these contexts, intuitive, fast, and involuntary forms of thought generate cognitive shortcuts which dominate decision-making (1, 61, 91, 92). Network science and complexity science expand the focus from individuals to the social interactions between individuals, enabling a comprehensive study of the properties of the entire complex adaptive human network (94–97). Such a network is based on the axiom that the whole is greater than the sum of its parts alone, suggesting emergent properties from groups, such as the formation of an organization's culture (94–97). The most highly connected individual (hub) or those individuals (bridges) that belong to multiple communities within the complex adaptive human network are often the most crucial individuals to facilitate the diffusion of evidence-based solutions (1–3, 61, 98, 99). Health systems are complex adaptive networks, where hubs may be senior frontline clinicians with numerous connections to other staff or, at a higher level, an entire health center may be considered a hub if it has a high number of institutional partners (100–103). These complex adaptive networks have predictable hierarchies of information exchange, as seen in Figure 1 (94–100).

In order to increase the demand for the adoption of health guidelines, like in the case of SCD, we propose Agile Storytelling as a framework for storytelling based on the principles of Agile Science. Integrating traditional storytelling with behavioral economics can contribute to the cognitive landscape by leveraging biases such as anchoring (creating a reference point for data), framing (presenting information in a specific context), affect (incorporating emotion into decision making), social proof (presenting the previous impact on others), and visual imagery (being able to visualize a scenario) to maximize story retention and behavior change (92, 93, 104–106). By mapping and understanding the connections between individuals and across hierarchies, Agile Storytelling is uniquely designed to leverage network properties and information dissemination pathways to maximize the impact of stories, thereby creating demand. In this paper, we describe how we used Agile Storytelling as a structured storytelling process to create local demand for the implementation and diffusion of evidence-based care services for people living with SCD in a large, statewide, integrated healthcare delivery network operating within the United States.

## 2 Methodology

Agile Storytelling is a two-phase process. The first phase converts evidence-based solutions into minimally viable stories, or stories with the most basic possible layout that can then be adapted to different audiences and contexts. The second phase tests such stories in a series of storytelling sprints, or rapid and iterative cycles of trial and error, within the local context of the targeted healthcare delivery organization.

The first phase works to build a minimally viable story based on an evidence-based solution to a specified health systems problem. The first step to developing a story is to find a narrative worth sharing, one that identifies instances of individual harm or an isolated instance of interventional success. This narrative can be derived from a personal situation or an experience within an impacted population. Upon doing so, the narrative must be converted into a story with struggle, drama, a villain, a hero, and a resolution. The narrative around the characters, particularly the hero and villain, are integral aspects of effective storytelling (107, 108). The struggle is the triggering event intended to capture the attention of listeners at the beginning of the story, appealing to salience biases through emotion and personal affect. The drama galvanizes or transforms listeners, translating the desired message into a comprehensive format. The villain is the person or concept at the center of the story, and the hero emerges as the winner from the struggle with the villain. The listener must relate to the hero. The attitude or behavior change of the hero throughout the story acts as a model for the listeners. The hero should experience hardships, change, overcome them, and walk away with valuable life lessons. The resolution of the story then presents these life lessons in an operationalized format, playing into the peak-end bias where the final items are most likely to be remembered. For a listener to buy into a story, the storyteller must already be committed. By illustrating the overlap between their passion and the listener's passions, the storyteller can transfer demand. The story should therefore be modified in accordance with the desired response by the intended population, whether it is knowledge translation, attitude change, or behavior change. This comes from knowing the background of and studying the audience deeply to highlight struggles the audience will empathize with, speak in rhetoric consistent with the in-group, and construct a hero who is personally relatable to audience members.

**Figure 2:**
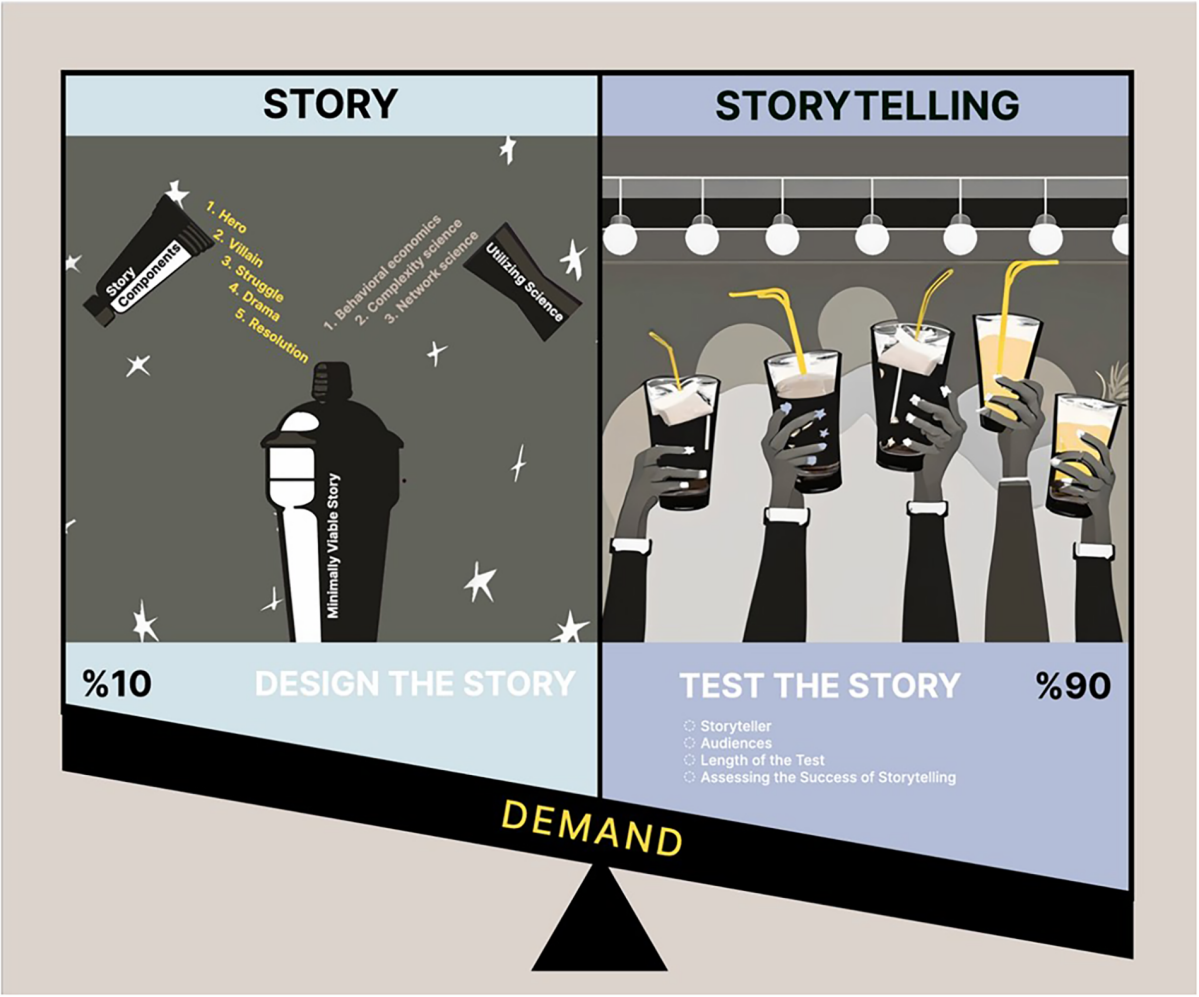
Agility balance between designing and testing a story.

The second phase tests the minimally viable story in a series of storytelling sprints within the local context of the targeted healthcare delivery system. After following these structured processes, the generated story is intended as a first draft and meant to be updated through iterative cycles of sprints to enhance the saliency and relevance of the story for specific audiences and contexts. Each sprint must include specified start and end dates, a method to collect information about the audience's response to the story, a storyteller to deliver the story, and time allocated to reflect on the results of the sprint. Storytelling can range from being entirely pre-scripted to fully improvisational, depending on the preference of the storyteller and knowledge about the intended audience. Improvisational storytelling can be personalized based on crowd reactions, updating in real time based on feedback gathered through deep observation. Storytelling must be conversational. Elements like dramatic pauses, voice inflection, illustrative diction, and metaphorical rhetoric are pivotal in capturing and retaining the audience's attention, conveying personal buy-in, and eliciting a memorable pitch. Multisensory components in storytelling –such as audio, pictures, or videos– can also enhance engagement and recall, so long as they are not overly distracting (109). Incorporating gamification in narratives can further improve knowledge translation, cooperation, and the real-world application of desired interventions or mindsets, which holds true in healthcare decision-making contexts (110). Modifications to the story, storyteller, or delivery are accepted or rejected based on pre-defined evaluation and termination plans for each sprint. The agility of the storytelling process comes from 90% of the time allocated for telling the story, and only 10% for planning, reflecting, and adjusting the story (see Figure 2) (1, 3, 61, 62, 78).

The opportunity to test the efficacy of Agile Storytelling to rapidly create demand and inspire change in accordance with evidence-based guidelines was presented in a large, statewide health system where a team sought to create a collaborative care based practice for the treatment of SCD.

## 3 Results—case study

The NHLBI and the American Society of Hematology recommend that health systems utilize a collaborative care model where a specific team is designated to provide care for sickle cell patients regardless of physical location (111–114). Multiple studies have concluded that across diverse health systems, as SCD patients continue to live longer, the need for collaborative care practices for SCD is increasing (115–117). A group within a cancer center in central Indiana, in the United States, attempted to adopt the NHLBI recommended collaborative care model for SCD, with the goal of having a sickle cell-specific social worker as the main care navigator for the collaborative care model. The team spent a year gathering data and reviewing the literature trying to convince the administration of the necessity for adopting the collaborative care model, including the need to hire a SCD-specific social worker as the care navigator. The group presented their findings over email and in dozens of meetings with supervisors, without any success. After receiving training in Agile Science, the team realized that they needed to secure demand from the leadership of the healthcare delivery system before it would be possible to create change. The team also recognized the only way to create demand for the project would be using Agile Storytelling.

The first step was to find the right story. One particularly emotional patient experience was selected to highlight the direct harms of the current system. The team selected the physician of the patient to be the storyteller, given their passion and dedication to the well-being of this patient. The physician messenger then converted one patient's experience into a minimally viable story with struggle, a villain, drama, and a resolution by making a SCD-specific social work position the hero. The team also had to map the complex adaptive human network for the cancer center facility to identify and adapt the story to the right target audience. The ideal target audience was the individual(s) with the authority to introduce a new social worker job position. The group surveyed the informal hierarchy, discussing with colleagues who might be the right individual to talk to. This endeavor proved not to be straightforward, as the cognitive bias of ego hindered many from recognizing when they lacked authority. The absence of a clear decision-making hierarchy, with roles distributed over groups of administrators, further complicated the process. The team isolated the organization's formal hierarchy by clicking through work charts to construct the official reporting structure and then modified this model with insight from the informal hierarchy. After many cycles of trial and error, the ideal target audience was found to be the executive director who all the social workers in the organization directly reported to. This individual was not only correctly positioned within the hierarchy to be a receptive audience, they also were a hub amongst the administrators who had the power to hire new workers and change organizational structures. The physician storyteller then sought to investigate the complexity surrounding the target individual. Transferring demand rests on recognizing competing priorities and appealing to common ones. The physician leveraged previous networking to recognize that the executive director was personally and authentically interested in reducing inequity and disparities in the cancer center. Zooming out, the team also considered organizational priorities and what drove organizational change: efficiency and an institutional commitment to excellence in patient care. The team experimented with different ways to modify the story to align patient needs, personal interests, and organizational priorities to best generate the investment of time, social, and financial capital. After running sprints with existing administrative connections, the team chose to appeal to emotional salience and peak-end behavioral principles.

*When I entered my morning clinic, there were already patients in the waiting room. I checked my schedule: it was going to be a busy day. My third patient that day had sickle cell disease. I had known her for many years. She was from an underserved community and was working full time but still didn*’*t have enough money for a car. She had previously had many bad experiences with the healthcare system leading to distrust towards many providers. I was one of the few doctors she trusted. I was usually able to bring a smile to her face and she knew I was committed to treating her disease and her pain. When I got into the room that day, my patient already had tears lining her cheeks. She told me she was in overwhelming pain, that it was affecting her everyday tasks and her job, and she was just so tired of it. She told me I wouldn*’*t understand the magnitude of her pain, I agreed. I began walking her through different options, adjusting medications, being admitted, running labs to make sure her blood counts were where they needed to be, but I knew she really just needed someone to care. I was already over the 30-minute timeslot for the follow-up visit, when a social worker came into the room. I was thrilled! This was exactly what my patient needed, someone to intervene in the crisis, empathize with her experience, and help her cope with her everyday experiences. I felt relief wash over me, this is what the system was designed to do, have multiple professionals attending to a patient*’*s needs holistically. I would be able to get back on schedule, to not keep my other patients waiting, while knowing my current patient was in good hands. Or so I thought. Much to my surprise the social worker walked briskly over to the patient, having her sign off on her transportation arrangements for the day, before turning and leaving. The social worker couldn*’*t have been in the room more than a minute or two. I was shocked, horrified, I knew the system had failed my patient and I knew she recognized that as well. She felt frustrated and helpless. More than anything else she just needed someone to listen, so I pulled up a chair. I spent another half hour consoling her and working through her current situation, even managing to get her to laugh. She hugged me before she left and told me ‘We need more people like you to help people like me.’ I knew what she meant and couldn*’*t agree more. If we had a designated social worker for patients with sickle cell disease, they would have the time to convey their full empathy and provide personalized resources, including long-term counseling. This experience could have been different, and by hiring a sickle cell specific social worker we can make sure it will be for all future patients treated by this health system with sickle cell disease*.

After telling this story just once to the target audience, a new SCD-specific social worker position was drafted in one week. No new data was shared, there was no change in authority, but one story was able to better demonstrate the need for change than a year's worth of statistics. The SCD social worker became a champion of SCD patients and was pivotal in the decision to employ a fully specialized collaborative care team for sickle cell disease in the health network (113, 118, 119). This ultimately increased the quality of care provided and reduced overall readmission rates, in line with national evidence-based best practice recommendations (38, 113, 118, 119).

## 4 Discussion

The case study used Agile Storytelling to create demand for an evidence-based care model for SCD. A minimally viable story was created and adapted to the intended audience. Sprints were executed to modify the story based on feedback that aimed to address multi-level priorities. And as a result, sharing the patient's experience facilitated the rapid hiring of a SCD-specific social worker. This approach created demand by aligning interests for reducing inequity and disparities while upholding excellence in patient care, along with emphasizing the need for a SCD-specific social worker in a memorable and emotive way. If the target audience for this change had been multiple different departments or across multiple organizations, the initial minimally viable story could have been customized to suit diverse audiences. Further, if the audience could not have been contacted directly, knowledge of the complex adaptive network could have been used to spread the story throughout communities of least resistance or bridges in order to reach the hub (97–99). Even if a story has worked before, this does not guarantee it will work in all communities or contexts, which is why sprints of minimally viable prototypes during story development are so valuable.

While the example in question took place in an urban center within the United States of America, there is a global need for increased adoption of SCD guidelines (27, 28, 42–44, 111–117). Given that narratives can be found in most cultures around the world, we believe this intervention has promising benefits for an international audience (48–53). Prior work suggests Agile Science methods and implementation science frameworks may be especially helpful in low- and middle-income country health systems, though more research in this field is recommended (101, 120–122). Since storytelling is already embedded in many cultures, this highlights a unique benefit over illustrative case reports or related designs; while they may similarly present an issue in an in-depth and real-life context, it may be more difficult to connect with a diverse audience (123). Further, illustrative case reports lack the discipline and structure of storytelling necessary for emphatic appeal, failing to minimize the separation between the messenger and the audience (54, 124, 125). Besides the Agile Storytelling process, the Medical Research Council has a model for storytelling guidance on developing complicated interventions (MRC Framework). The MRC Framework divides complex intervention research into four phases: development/identification, feasibility, evaluation, and implementation (126). Each phase has a standard set of core elements that engage stakeholders and identify uncertain elements to revisit throughout the research process (126). Similarly, the Capability, Opportunity, Motivation to Behavior (COM-B) model combines capability and opportunity to influence motivation and behavior (127–129). Both frameworks differ from Agile Storytelling because they lack the iterative cycles of testing to find the right story, share personal passion, and incorporate behavioral principles. Agile Storytelling specifies developing a story with heroes and villains relatable to the target audience to aid in transferring demand. Also, by mapping the complex adaptive human network in which the story is told, one can strategically align multi-level priorities and broadly diffuse the message. This provides unique advantages compared with existing storytelling methodologies. Programs based on Agile Science have been found to create widespread change across diverse initiatives, and Agile change conductors trained in such processes are consistently able to implement evidence-based health interventions (1, 61, 100, 130, 131). Agile Storytelling overcomes data-to-practice translational gaps and facilitates the adoption of evidence-based healthcare guidelines.

Despite Agile Storytelling being effective in bringing about change, it faces challenges for proper utilization and generalizability. Being able to storytell and deeply understand an audience takes practice and can be more difficult in large, complex organizations or across diverse cultures. Another challenge is contacting those with the authority to make decisions; even when aware of the appropriate audience, overcoming hierarchical constraints to schedule a meeting is not always feasible. Most employees at a hospital rarely interact with administrative leaders, making it difficult to inquire about the priorities and perspectives of administrators on various topics. It is especially hard to garner enough psychological safety to receive constructive criticism, meaning that sprint feedback relies primarily on observation. While case study approaches to storytelling are beneficial for providing detailed examples, the small sample size (*n-*of-1) does hinder the representativeness and generalizability of reports. This prevents concrete conclusions and necessitates future research applying the same methodology to different geographical and sociocultural contexts. It is also worth exploring digital or multisensory mediums for Agile Storytelling, an aspect not present in the current case study, given the potential to expand the impact of storytelling without losing personalization. Finally, a limitation to presenting Agile Storytelling as a case study is that outcomes are expected to vary across audiences. For example, certain decisions presented may be less applicable in organizations without a formal hierarchy, in cases of competing institutional priorities, or in cultures with different display rules for expressing emotion. Furthermore, it may be essential to tell a story many times to dissimilar audiences, rather than to one individual with the ability to broadly diffuse information, before the desired changes are made. This prevents the explicit identification of an optimal number of times to tell a story or individuals to tell a story to in any storytelling framework. Nevertheless, traditional storytelling does not have the purpose of creating demand, does not consider the importance of the storyteller, and does not understand the target audience as existing within a complex adaptive human network. Traditional storytelling divests all the energy and capital into designing the story, failing to test the minimally viable story within the targeted network, limitations which Agile Storytelling was designed to overcome.

In conclusion, data and statistics alone do not appeal to cognitive heuristics with the emotional affect that stories do, making Agile Storytelling a promising avenue for creating demand for the adoption of evidence-based practices and breaking through existing health barriers across diverse populations.

## Data Availability

The raw data supporting the conclusions of this article will be made available by the authors, without undue reservation.
